# The effect of foot bath on sleep quality in the elderly: a systematic review

**DOI:** 10.1186/s12877-023-04590-x

**Published:** 2024-02-26

**Authors:** Khadijeh Nasiri, Mohammad Shriniy, Nazila Javadi Pashaki, Vahideh Aghamohammadi, Solmaz Saeidi, Maryam Mirzaee, Mostafa Soodmand, Esmail Najafi

**Affiliations:** 1 Department of Medical-Surgical Nursing, Guilan University of Medical Sciences, Guilan, Iran; 2 Student Research Committee, Department of Nursing, Khalkhal University of Medical Sciences, Khalkhal, Iran; 3 Department of Medical-Surgical Nursing, Guilan University of Medical Sciences, Guilan, Iran; 4 Department of Nutrition, Khalkhal University of Medical Sciences, Khalkhal, Iran; 5 Department of Public Health, Khalkhal University of Medical Sciences, Khalkhal, Iran

**Keywords:** Sleep quality, Elderly, Foot bath, Sleep

## Abstract

**Introduction:**

Population aging is a problem that has affected most countries in the world. Poor-quality sleep is a common complaint among the elderly. Foot baths are a method of heat therapy and are performed as an independent nursing care in different departments. The present study was conducted with the aim of investigating the effects of foot baths with spa on improving the sleep quality of the elderly.

**Methods:**

This research is a systematic review. We systematically searched six databases, including Google Scholar, PubMed, Web of Science, Scopus, Embase, and the World Health Organization databases, to retrieve the related articles based on the keywords used in our search strategy from 2010 to March 2023.

**Result:**

Finally, 10 articles were included in this study. All studies were randomized controlled trial (RCTs) and semi-experimental. In all 9 studies, the positive effects of the foot bath were reported. In 9 studies, the effect of foot baths with water above 40 degrees Celsius was reported. The PSQR questionnaire was used in most of the studies.

**Conclusion:**

The total findings of this study showed that due to the high prevalence of sleep problems in the elderly, foot baths with warm water can be used as an easy, simple, and safe nursing intervention to improve sleep quality. Therefore, it can be used in nursing homes and hospitals. It is also a non-pharmacological and inexpensive nursing intervention that can be implemented by the elderly themselves after training by community health nurses.

## Introduction

Population aging is a problem that has affected most countries in the world [1]. According to the statements of the Organization for Economic Co-operation and Development (OECD), it is expected that the population of individuals aged 65 and older will increase by 25.1% in member countries by 2050 [2]. In today’s world, where the population is rapidly aging, issues concerning old age are receiving great attention and importance [3]. Poor-quality sleep ranks as the third most common complaint among the elderly, following headaches and digestive disorders [4]. Good and sufficient sleep is one of the basic needs for human health, and the National Sleep Foundation states that the adequate amount of sleep for adults is 7 to 9 h per night [5]. The results of the research conducted by Trabelsi et al. (2021) showed that more than half of the elderly population suffer from sleep disorders, and this decrease in sleep quality is considered one of the main causes of other physical and mental problems [6]. Sleep is a physiological need, and its adequate and appropriate amount plays an important role in maintaining health and optimal physical and cognitive performance. Both the quantity and quality of sleep play an important role in a person’s performance the following day and their quality of life [7]. Complications arising from chronic insomnia encompass depression, daytime fatigue, irritability, impaired daily functioning, and issues with cardiovascular and digestive health, in addition to adversely impacting overall quality of life [8, 9]. Furthermore, research indicates that insufficient sleep is linked to a myriad of diseases and health disorders, including an elevated risk of obesity, metabolic disorders, depression, suicide, substance abuse, post-traumatic stress disorder, accidents, and injuries [10–13]. Sleep plays a crucial role in maintaining biochemical, hormonal, and metabolic functions, sustaining overall body function, and ensuring physiological and psychological equilibrium, alongside homeostatic adaptation [14]. The significance of sleep has deep roots in nursing history and Florence Nightingale’s holistic perspective, which recognizes sleep as a fundamental human need [15]. Consequently, nurses, as integral members of the healthcare team, hold a vital role in addressing sleep-related issues [16]. Diverse nursing interventions have been explored to enhance sleep quality, encompassing massage therapy [17], aromatherapy [18], herbal medicines [19], heat therapy [20] and foot bath [21]. A foot bath, considered a form of heat therapy, is implemented independently in various departments [22]. Widely accepted and popular in many Asian countries, foot baths have been investigated and confirmed for their positive effects since the 1960s [23]. Bathing feet with warm water before going to bed is a widely used method that improves the quality of sleep [24]. There is a functional relationship between the circadian rhythm of skin temperature and central body temperature (rectal temperature) within the sleep-wake cycle. The circadian rhythm of core body temperature is characterized by a relatively low temperature during sleep and a relatively high temperature during wakefulness. A negative relationship exists between central body temperature and the desire to sleep, with the onset of sleep facilitated when the central body temperature reaches its lowest level [25]. Core body temperature begins to rise in the morning, reaching its maximum in the afternoon. The decrease in rectal temperature before and during sleep is associated with the dilation of peripheral vessels and is attributed to the conduction of heat from the center to the periphery of the body [26, 27]. Numerous studies have investigated the effect of foot baths on improving sleep quality. Aghamohammadi and colleagues conducted a study showing that foot baths with warm water improve the quality of sleep in menopausal women [28]. In another trial involving adults, the results indicated a positive effect of foot baths on improving post-operative sleep quality [29]. A pilot study demonstrated that foot baths enhance peripheral blood circulation [30]. In contrast to the study by Liao et al., foot baths did not affect the quality of sleep [31]. Additionally, in another study conducted on patients with acute coronary syndrome, it was reported that while foot baths did not improve sleep quality in all patients, they did reduce the number of patients with severe sleep disorders [32]. In a separate study, foot baths improved the sleep quality of elderly people living in nursing homes [33]. Due to the ambiguity in the effectiveness and the lack of an objective determination of the effects of foot baths on improving the sleep quality of the elderly, there is a need for a systematic review of the studies conducted in this field. Therefore, the present study was conducted with the aim of investigating the effects of foot baths with a spa on improving the sleep quality of the elderly.

## Methods

### Study design

This research constitutes a systematic review. We systematically searched six databases, namely Google Scholar, PubMed, Web of Science, Scopus, Embase, and the World Health Organization databases, to retrieve relevant articles based on the keywords used in our search strategy from 2010 to March 2023. Screening occurred in two stages, with two researchers involved. During the first stage, the title and abstract were checked, and in the second stage, the full-text screening process was carried out. Any remaining discrepancies were addressed by a third researcher. Data extraction and summarization of the included studies were performed by two researchers. The reporting method in this study adhered to the PRISMA (Preferred Reporting Items for Systematic Reviews and Meta-Analysis) checklist [34].

### Search strategy

Search strategy included:

1. " Foot Bath “OR “Warm Water Foot Bath " OR " Warm Foot Bath” OR “Water Foot Bath Therapy “[Title/abstract/keywords].
2. “Sleep Quality” OR “sleep” OR “Quality of Sleep” [Title/abstract/keywords].
3. " Elderly “OR “Elderly People” OR " older adults” OR " old people " [Title/abstract/keywords].
4. [A], [B] and [C].

### Inclusion and exclusion criteria

We included all English clinical trial and semi-experimental studies conducted with the aim of investigating the effect of foot baths on the sleep quality of the elderly. The exclusion criteria were as follows:

F. (1) Review articles, letters to the editors, or other studies without original data.
G. (2) Ongoing studies.
H. (3) Studies irrelevant to the aims, settings, and design of this research.
I. (4) Abstracts, conference abstracts, errata, or other studies lacking full texts, and studies whose full text was not in English.

### Quality assessment

The quality of the articles was evaluated using the Jadad scale, which consists of five questions related to the experimental nature of the study, the randomization method, the possibility of bias, blinding, and patient follow-up. The maximum evaluation score is 5, and the minimum score is 0. Articles with a score of 3 or more are considered to have appropriate methodology. It is important to note that the results were analyzed qualitatively [35].

### Data extraction

**Fig. 1:**
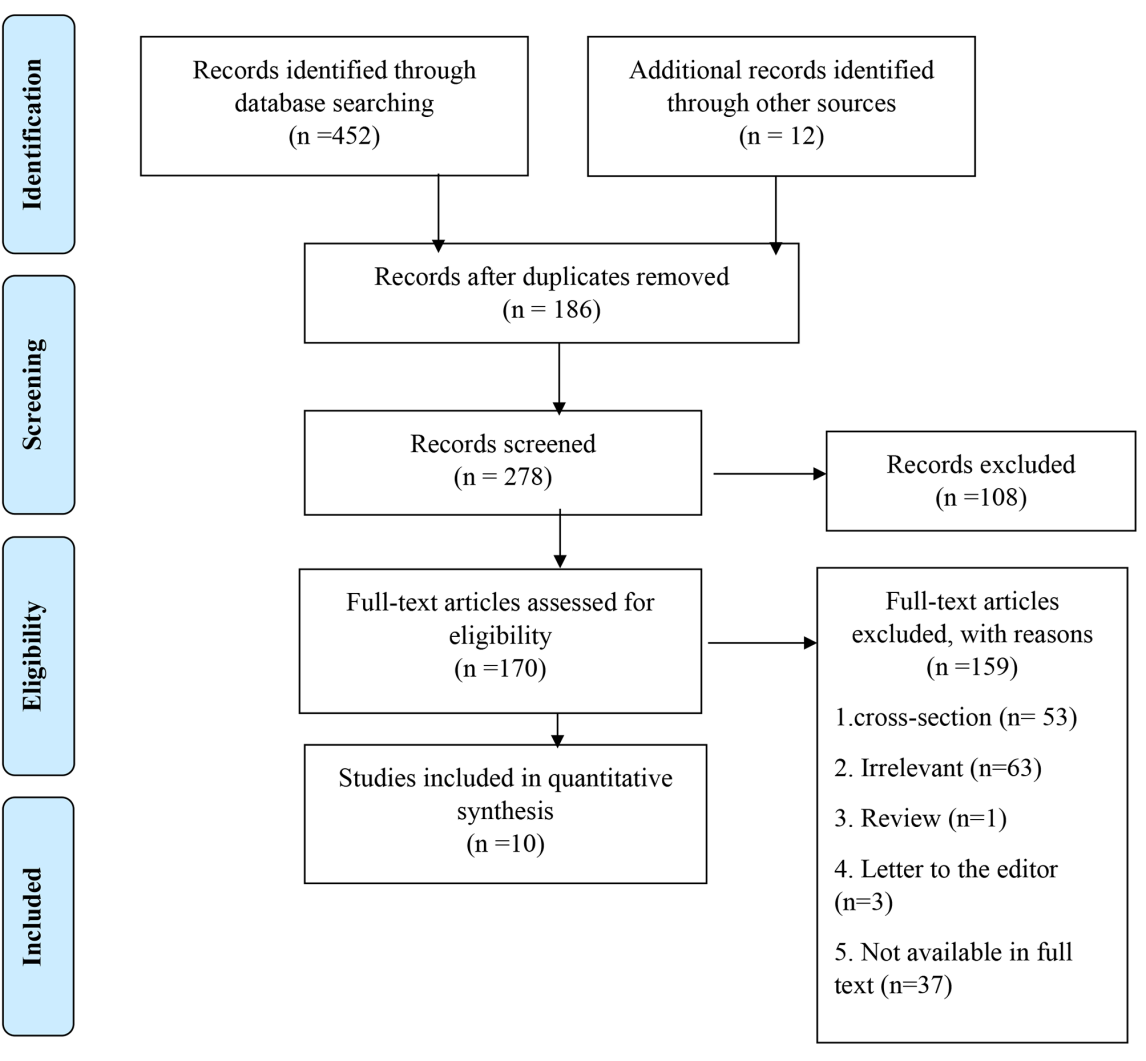
PRISMA 2020 Flow Diagram of the Selection Process of the Systematic Reviews

The authors’ names, publication date, type of study, gender, sample size, control group, water temperature, time before bedtime, duration of foot bath, instrument, and results of the studies were recorded in an information sheet. Additionally, the full text of selected articles was read, and the key findings are summarized in the table. In this systematic review, 452 documents were identified. After a primary review of retrieved articles, 186 duplicates were removed, and the title and abstract of the remaining articles were reviewed. One hundred and eight articles were excluded after applying the selection criteria. One hundred and seventy full-text articles were assessed for eligibility, with 159 of them being excluded due to irrelevance, being reviews, letters to the editor, or not being original articles. Ultimately, 10 articles met the inclusion criteria and were included in the final review (Fig. 1).

**Table 1:** Effect of foot bath on sleep quality in elderly

Author (y)	Type of study	Gender (n)	Control group	Water temp (^o^ c)	Time before bed time	Duration of foot bath	Duration of each session	instrument	result
Seyyedrasooli (2013) [36]	RCT	Male (46)	yes	40–42	1 h before Bed time	6 week	20 min	PSQI	Foot bath improve sleep quality (3 Points)
Liao (2013) [31]	RCT	Both (43)	yes	40 ± 0.5	2 h before Bed time	3 night	20 min	PSQI & Polysom-nography	No significant Foot bath unimproved sleep quality
Kim (2016) [37]	Quasi-experimental	Both (30)	yes	40	-	4 week	30 min	ATG	Foot bath improve sleep quality
Ong (2018) [38]	RCT	Female (16)	No	40	1 h before Bed time	2 night in week	20 min	PSQI	Foot bath improve sleep quality (4.82 Points (
Malarvizhi (2019) [33]	Quasi experimental	Both (60)	yes	43–46	10 min Before bed time	7 days	15 min	MGSQS^*^	Foot bath improve sleep quality (3.4 Point)
Puspitosari (2021) [39]	Pre-experimental	Both (17)	No	35	10 min Before bed time	3 times per week for 4 week	10 min	PSQI	Foot bath improve sleep quality (2.35 Points)
Armat (2021) [40]	RCT	Both (45)	Yes	37 and 40	1 h before Bed time	4 week Each night	10 min	PSQI	both 37 (^o^ c) and 40 (^o^ c) improved sleep quality
Das (2021) [41]	Quasi-experimental	Both (40)	yes	40–46	Each evening.	5 day	10–30 min	MGSQS	Foot bath improve sleep quality (3.4 Point)
Ghosh (2021) [42]	Quasi-experimental	Both (60)	yes	40 ± 2	twice in day	7 day	15 min	MGSQS	Foot bath improve sleep quality (7.4 Point)
Güven (2022) [43]	RCT	Both (50)	yes	40 ± 2	1 h before Bed time	6 week	30 min	PSQI	Foot bath improve sleep quality

* Modified Groningen Sleep Quality Scale.

** Veran Snyder-Halpern.

## Result

This systematic review investigated 10 clinical trial and semi-experimental studies, involving a total of 407 elderly participants across intervention and control groups. The findings from 9 of these studies indicated that foot baths were associated with an improvement in the sleep quality of the elderly [33, 36–43]. 8 studies had a control group [31, 33, 36, 37, 40, 41, 43].In most studies, water with a temperature higher than 40 **(**^**o**^**c)** was used for foot bath [31, 33, 36–38, 41–43] and in two studies, a temperature of 35–40 **(**^**o**^**c)** was used [39, 40]. The timing of foot baths was documented in four studies, with one hour before sleep reported in studies [36, 38, 40, 43], 10 min before sleep in two studies [33, 39], two hours before sleep in one study [31], twice a day in one study [42] and in the evenings in one study [41]. Additionally, one study did not specify the timing of foot baths [37]. The duration of foot baths varied across studies, ranging from three nights [31], four weeks [37, 39, 40], five days [41], seven days [33, 42] and to six weeks [36, 43]. The study findings indicated that the duration of foot baths in each session was reported as 20 min in three studies [31, 36, 38], 30 min (2 studies) [37, 43], 10 min (2 studies) [39, 40], 15 min (2 studies) [33, 42] and 10–30 min (1 study) [41] was reported. Furthermore, various tools were employed to assess sleep quality across studies, with the Pittsburgh Sleep Quality Index (PSQI) used in six studies [31, 36, 38–40, 43], MGSQS used in 3 studies [33, 41, 42] and ATG tool was used in 1 study [37]. The results are presented in Table 1.

## Discussion

The primary objective of this systematic review was to examine the impact of foot baths on the sleep quality of elderly individuals. The collective results of this investigation revealed a positive influence of foot baths on enhancing the sleep quality among the elderly. The studies assessed in this systematic review comprised randomized controlled trials (RCT) and quasi-experimental designs, with a significant proportion including a control group. This inclusion of control groups enhances the credibility and validity of the study outcomes. The findings of this research demonstrated that, in 80% of the conducted studies, water temperatures of 40 degrees Celsius and higher were associated with improved sleep quality in the elderly, aligning with the results observed in other studies [44, 45]. Physiologically, it has been established that foot baths are linked to sleep by influencing core body temperature. A negative correlation exists between central body temperature and sleep, and reducing central body temperature facilitates the sleep process, leading to an improvement in sleep quality [46]. In this context, ancient Greek philosophers’ studies have suggested a connection between the sleep state and the redirection of blood from peripheral vessels to larger vessels [47]. Despite the prevalent focus on temperatures of 40 degrees Celsius and above in most studies, two studies within this systematic review demonstrated that foot baths with water temperatures ranging from 35 to 40 degrees Celsius also positively impacted the sleep quality of the elderly [40]. The outcomes of the current study align with those of a systematic review and meta-analysis conducted by Haghayegh et al. in 2019 [48]. In their research, all 13 reviewed studies utilized temperatures of 40 degrees Celsius and above to enhance sleep quality. This systematic review’s results revealed that only the study by Liao et al. in 2013 did not confirm the foot bath’s effect on sleep quality. The short duration of the foot bath and the utilization of objective methods (polysomnography) in addition to subjective methods seem to be contributing factors to the contradictory results observed in this study compared to others [31]. Furthermore, the systematic review’s findings highlighted the prevalent use of the PSQI questionnaire as the primary tool for assessing sleep quality. Developed by Buysse et al. in 1989, this questionnaire aids in measuring sleep quality and diagnosing sleep disorders [49]. Across all studies employing this tool to evaluate the impact of foot baths, the intervention consistently improved sleep quality by a minimum of 2 points. These results are in harmony with studies conducted on non-elderly populations [24, 28]. The similar findings may be attributed to the PSQI questionnaire’s self-report nature, focusing solely on the subjective aspect of sleep quality. Notably, the only study mentioned in this systematic review that deviated from this trend was the clinical trial conducted by Liao et al. (2013), which, alongside the PSQI self-report tool, incorporated polysomnography to assess sleep quality [31]. Another commonly utilized tool in studies exploring the impact of foot baths on the sleep quality of the elderly is the MGSQS questionnaire. This questionnaire assesses various facets of sleep and has been employed across diverse studies to evaluate sleep quality. When investigating the foot bath’s effect on enhancing sleep quality, this tool consistently reveals the positive impact of the intervention. The congruent and positive outcomes from both tools serve to validate the efficacy of foot bath interventions as a safe and healthful method.

The results of this systematic review indicate that a foot bath lasting between 10 and 30 min, conducted for the elderly from ten minutes to two hours before sleep, proves effective in enhancing sleep quality. This finding aligns with results from other studies [24, 28, 45]. Additionally, the systematic review study and meta-analysis conducted by Haghayegh et al. demonstrated variations in the timing of foot bath interventions, ranging from immediately before bedtime to approximately 6 h before bedtime. Haghayegh suggests that taking a foot bath two or more hours before bedtime may induce drowsiness but might not significantly enhance sleep quality. However, the authors of this study emphasize the need for further research to determine the optimal timing and duration of foot bath interventions [48]. Other studies examining the effect of foot baths on sleep quality propose a suggestion of one hour before the usual bedtime for this intervention [24, 28, 50].

## Conclusion

The overall results of this study indicate that, given the widespread occurrence of sleep issues among the elderly, the utilization of warm water foot baths can serve as a straightforward, uncomplicated, and safe nursing intervention for enhancing sleep quality. Consequently, this intervention can find practical application in nursing homes and hospitals. Furthermore, being a non-pharmacological and cost-effective nursing intervention, it can be easily adopted by the elderly themselves after receiving guidance from community health nurses.

## Data Availability

The data that support the findings of this study are available from the corresponding author, upon reasonable request.
